# Scott Co. Med. Sc’y.

**Published:** 1857-06

**Authors:** J. J. Tomson

**Affiliations:** Sec’y.


### Scott County Medical Society.

Agreeably to notice publicly given, a number of medical gentlemen in the County of Scott, Iowa, met at the office of Drs. Witherwax & Carter, in Davenport, on Saturday, October 18th, 1856, at 2, P. M., for the purpose of organizing a Medical Society.

Present—Drs. Alder, Baker, Carpenter Senior, Carpenter Junior, Maxwell, Carter, Pelton, Saunders, Thistle and Tomson.

Dr. Jas. Thistle was called to the Chair, and Dr. J. J. Tomsom was appointed Secretary.

The object of the meeting having been stated, on motion the following committees were selected by the chair and instructed to report at a subsequent meeting, viz:

On Constitution and By-Laws: Drs. Alder, Baker and Pelton.

On Code of Ethics: Drs. Maxwell, Carpenter, Sr., and Carter.

On Fee Bill: Drs. Line, Carpenter, Jr., and Tomson.

Then adjourned to meet at the office of Drs. Fountain & Alder, on Tuesday, Oct 28th, at 2 o'clock, P. M.

At the time and place adjourned to, the meeting again convened.

Present—Drs. Alder, Baker, Carpenter, Sr., Carpenter, Jr., Carter, Line, Maxwell, Marcelis, O'Reardon, Pelton, Saunders, Thistle and Tomson.

The Chairman called the meeting to order, and the minutes of the last meeting were read by the Secretary and approved.

Dr. Alder, from the committee on Constitution and By-Laws, made a report, which, after being strictly amended, was unanimously adopted.

[This report provides that the Society shall be subordinate to the Iowa State Medical Society; that it shall consist of such physicians and surgeons, residing in the county of Scott, as are regular members of the profession, that its officers shall be a President, Vice President, Secretary, Treasurer, and three Censors, all to be elected by ballot; and that its annual meetings shall be on the last Tuesday of October, and the three other meetings on the last Tuesdays of January, April and July, respectively, in the city of Davenport.]

Mr. Maxwell, from the committee on Code of Ethics, reported in favor of that which had been adopted by the "American Medical Association," which report was unanimously approved.

An election was then entered into for officers for the ensuing year with the following result, viz :

Dr. Egbert S. Barrows, President.

Dr. Lyman Carpenter, Vice-President.

Dr. J. J. Tomson, Secretary.

Dr. Jas. Thistle, Treasurer.

Drs. Th. J. Saunders, Jno. M. Adeer, and J. W. H. Baker, Censors.

Ordered, That the Secretary and Treasurer be authorized to procure such books, &c., as the Secretary may require. Also that a special meeting be held at the hall of the "Young Men's Literary Association," on Tuesday, Nov. 11th, at 2 o'clock, P. M., for the purpose of signing Constitution and By-Laws, and for the transaction of such other business as may come before the society.

The meeting then adjourned. J. J. Tomson, Sec'y.

